# Comprehensive characterization of genes associated with the TP53 signal transduction pathway in various tumors

**DOI:** 10.1007/s11010-017-2977-1

**Published:** 2017-03-03

**Authors:** Shumpei Ohnami, Keiichi Ohshima, Takeshi Nagashima, Kenichi Urakami, Yuji Shimoda, Junko Saito, Akane Naruoka, Keiichi Hatakeyama, Tohru Mochizuki, Masakuni Serizawa, Sumiko Ohnami, Masatoshi Kusuhara, Ken Yamaguchi

**Affiliations:** 1 Cancer Diagnostics Research Division, Shizuoka Cancer Center Research Institute, 1007 Shimonagakubo, Nagaizumi-cho, Sunto-gun, Shizuoka, 411-8777 Japan; 2 Medical Genetics Division, Shizuoka Cancer Center Research Institute, Shizuoka, Japan; 3 SRL Inc, Tokyo, Japan; 4 Drug Discovery and Development Division, Shizuoka Cancer Center Research Institute, Shizuoka, Japan; 5 Regional Resources Division, Shizuoka Cancer Center Research Institute, Shizuoka, Japan; 6 Shizuoka Cancer Center, Shizuoka, Japan

**Keywords:** Cancer, Gene expression profiling, Japanese population, TP53 pathway, Whole exome sequencing

## Abstract

The TP53 signal transduction pathway is an attractive target for cancer treatments. In this study, we conducted a comprehensive molecular evaluation of 907 patients with cancer in Japan to identify genomic alterations in the TP53 pathway. *TP53* mutations were frequently detected in many cancers, except melanoma, thymic tumors, gastrointestinal stromal tumors, and renal cancers. The frequencies of non-synonymous single nucleotide variants (SNVs) in the *TP53* family members *TP63* and *TP73* were relatively low, although genes with increased frequencies of SNVs were as follows: *PTEN* (11.7%) in breast cancer, *CDKN2A* (11.1 and 9.6%) in pancreas and head and neck cancers, and *ATM* (18.0 and 11.1%) in liver and esophageal cancers. *MDM2* expression was decreased or increased in patients with mutant or wild-type *TP53*, respectively. *CDKN1A* expression was increased with mutant *TP53* in head and neck cancers. Moreover, *TP63* overexpression was characteristically observed in squamous cell carcinomas of the lung, esophagus, and head and neck region. Additionally, overexpression of *TP63* and *TP73* was frequently observed in thymomas. Our results reveal a spectrum of genomic alterations in the TP53 pathway that is characteristic of many tumor types, and these data may be useful in the trials of targeted therapies.

**Electronic supplementary material:**

The online version of this article (doi:10.1007/s11010-017-2977-1) contains supplementary material, which is available to authorized users.

## Introduction

Genome sequencing is an essential tool for cancer research that leads to important biological discoveries and allows for the systematic classification of mutations based on cellular signal transduction pathways [1, 2]. Furthermore, data accumulated from studies using tumor tissues of patients with cancer has led to the identification of somatic alterations in many cancer-related genes [3, 4]. The *TP53* gene encodes a tumor suppressor and frequently undergoes somatic mutation in tumor cells [5]. A database of *TP53* mutations is available [6, 7], and there are detailed data regarding the functional activities of TP53 mutants.

TP53 mediates diverse cellular functions, including the response to DNA damage and induction of cell cycle arrest, cellular senescence, autophagy, and apoptosis [8–10]. Additionally, TP53 can regulate the cellular metabolism [11], inhibit stem cell self-renewal, and control the reprograming of differentiated cells into stem cells [8]. TP53 has also been shown to mediate tumor metastasis and invasion [12]. The disruption of signaling pathways that activate TP53 play an important role in tumor progression. Although *TP53* knockout mice develop normally, their susceptibility to cancers is higher than wild-type *TP53* animals [13]. *TP53* germline mutations in humans are associated with increased susceptibility to cancer and an earlier age of onset compared to *TP53* wild-type controls [14]. Li-Fraumeni syndrome is a rare, inherited, and highly penetrant disorder that predisposes individuals to cancer. This syndrome is characterized by autosomal dominant *TP53* germline mutation [15]. Thus, exploiting the tumor suppressor function of TP53 and the high frequencies of *TP53* mutations in cancer tissues represents an appealing therapeutic strategy for developing cancer treatments. However, despite numerous attempts to target the TP53 pathway [16, 17], there are currently no treatments available in the clinic [5].

TP53 activity is regulated by the E3 ubiquitin protein ligase and proto-oncoprotein murine double minute 2 (MDM2) and by post-translational modifications, such as phosphorylation and acetylation. MDM2 inhibits TP53 transcriptional activity by binding to the *N*-terminal domain of TP53, which leads to downregulation of the TP53 pathway [18]. Overexpression of *MDM2* in mice revealed a TP53-independent role in tumorigenesis [19], and *MDM2* overexpression or amplification occurs in many human cancers and contributes to oncogenesis [20, 21]. Previous studies have demonstrated that inhibiting MDM2-TP53 binding in xenograft models restores TP53 function and can inhibit tumor cell proliferation and induce apoptosis [22]. However, the data indicate that the mechanisms underlying these effects are associated with the more complex regulation of MDM2 expression. Although many TP53-associated molecules play important roles in regulating *TP53* transcription [8, 23], the regulatory mechanisms underlying its activation *in vivo* have not been fully elucidated. In this study, we present a comprehensive analysis of genomic alterations that are associated with the TP53 pathway in various tumors in a Japanese population. We analyzed tumor tissues and adjacent normal tissues and blood samples to identify tumor-specific somatic mutations. We anticipate that this comprehensive analysis will lead to the development of individualized treatment strategies.

## Materials and methods

### Subjects

The Shizuoka Cancer Center (Shizuoka, Japan) launched Project HOPE in late January 2014. The project objective is to improve cancer medicine [24]. As a component of this project, we performed whole exome sequencing (WES) using blood samples and fresh surgical specimens. We then conducted comprehensive analyses of gene expression using matched tumor and adjacent normal tissues from each patient. Tumor-specific single nucleotide variants (SNVs) were determined by comparing tumor tissue with blood cell data from the same patient. The characteristics of the subjects are summarized in Table 1, and the detailed histpathological characteristics are presented in Supplementary Table 1. The research plan was designed according to the revised Ethical Guidelines for Human Genome/Gene Analysis Research in Japan (http://www.lifescience.mext.go.jp/files/pdf/n1115_01.pdf) and was approved by the Institutional Review Board of the Shizuoka Cancer Center. All patients provided written informed consent.

**Table 1:** Patient characteristics

Cancer types	*n*	Age	Sex		Diabetes			Smoking Status			Drinking Status		
		Mean ± SD	Male	Female	Nondiabetic	Diabetic	Unknown	Non-smoker	Smoker^a^	Unknown	Non-drinker	Drinker^b^	Unknown
Stomach	116	71.1± 9.3	82	34	94	19	3	34	82	0	22	56	38
Lung	176	68.6 ± 9.9	114	62	145	29	2	50	126	0	16	85	75
Colorectum	311	66.0 ± 11.3	184	127	260	46	5	122	184	5	51	175	85
Breast	60	57.3 ± 13.0	0	60	58	2	0	46	13	1	19	21	20
Liver	61	70.3± 8.7	48	13	48	12	1	14	46	1	12	44	5
Head and neck	73	62.5 ± 14.8	48	25	63	9	1	25	48	0	11	36	26
Pancreas	18	67.3 ± 15.5	11	7	14	4	0	6	11	1	4	9	5
Kidney	13	65.3 ± 13.5	7	6	12	1	0	5	8	0	2	8	3
Esophagus	18	66.5 ± 8.9	15	3	15	2	1	5	13	0	2	10	6
Uterus	12	55.3 ± 12.8	0	12	12	0	0	11	1	0	0	4	8
Sarcoma	16	45.4 ± 20.4	11	5	16	0	0	8	8	0	3	8	5
Gist	9	70.4 ± 11.0	6	3	7	2	0	6	3	0	1	4	4
Melanoma	5	61.6 ± 16.9	3	2	4	1	0	4	1	0	1	2	2
Thymus	6	63.0 ± 9.0	1	5	5	1	0	4	2	0	0	2	4
Ovary	4	57.5 ± 14.6	0	4	4	0	0	4	0	0	0	3	1
Skin	3	52.0 ± 14.8	1	2	3	0	0	1	2	0	0	1	2
Brain	3	58.3 ± 8.1	2	1	3	0	0	1	2	0	0	2	1
Bile duct	2	72.0 ± 8.5	1	1	2	0	0	1	1	0	0	2	0
Gallbladder	1	71	0	1	1	0	0	0	1	0	0	0	1
Total	907	65.5 ± 13.0	534	373	766	128	13	347	552	8	144	472	291

^a^Smoker; past or current

^b^Drinker; occasional or regular

### DNA preparation

We obtained blood and tumor samples from 907 patients with cancer at the time of surgery. Surgeries were performed at the Shizuoka Cancer Center Hospital between January 2014 and March 2015. Sample genomic DNA was extracted from whole blood and tumor tissues using a QIAamp DNA Mini kit (Qiagen, Hilden, Germany). DNA was quantified using a Nanodrop spectrophotometer (Thermo Fisher Scientific, Wilmington, DE, USA) and a Qubit 2.0 fluorometer (Thermo Fisher Scientific). AcroMetrix Oncology Hotspot Control DNA (Thermo Fisher Scientific) was used as the standard.

### RNA preparation

Fresh tumor and adjacent normal tissue were soaked in RNAlater reagent (Qiagen). The total RNA was then isolated and purified using an RNeasy Mini kit (Qiagen) according to the manufacturer’s protocol. Total RNA was analyzed using a Nanodrop 2000 spectrophotometer (Thermo Fisher Scientific) and gel electrophoresis. The RNA quality was evaluated using gel electrophoresis and the A_260_/A_280_ value. The RNA integrity number (RIN) [25] was determined using an Agilent 2100 Bioanalyzer (Agilent Technologies, Santa Clara, CA, USA). We used RNA samples with an A_260_/A_280_ > 1.8 and a RIN > 6.0 for gene expression analysis.

### Whole exome sequencing (WES)

We performed WES using an Ion Proton System equipped with a PI chip V2 together with an AmpliSeq Exome kit (Thermo Fisher Scientific) [26]. Briefly, 100 ng each of tumor and matched blood cell DNA was used for target amplification with the following protocol: 99 °C for 2 min, followed by 10 cycles at 95 °C for 15 s and 60 °C for 16 min, and a final hold at 10 °C. The incorporated primer sequences were partially digested using FuPa Reagent (Thermo Fisher Scientific). Ion Torrent Proton adapters were ligated to the amplicons at 22 °C for 30 min and then at 72 °C for 10 min. The amplicon library was purified using Agencourt AMPure XP Beads (Thermo Fisher Scientific). The library DNA was quantified by qRT-PCR, and 7 pM library DNA was used for sequencing. The sequencing data were aligned to the human reference genome (assembly GRCh37/hg19) and were quality trimmed using Ion Torrent Suite version 4.2 (Thermo Fisher Scientific). The mutations were visualized using the Integrative Genomics Viewer [27] and were validated using Sanger sequencing or pyrosequencing.

### Validation of somatic mutations using deep sequencing of the Custom Cancer Panel (CCP)

The candidate mutations identified by WES were validated using the Ion Torrent PGM AmpliSeq Custom Panel (Themo Fisher Scientific) for 409 target genes (the target genes are available at https://www.thermofisher.com). We used a 200-bp standard DNA option to design the AmpliSeq primers. Sample DNA was diluted to 10 ng/µL, and 1 µL was used to prepare the amplicon library according to the manufacturer’s protocol (Themo Fisher Scientific). The target sequences were amplified using the customized primers and were then partially digested. The adapters and barcodes were ligated to the amplicons, which were then purified using the Agencourt AMPure XP reagent (Thermo Fisher Scientific). The libraries were sequenced using the same method described above for WES.

### Comprehensive gene expression analysis using a DNA microarray

Cyanin-3 (Cy3)-labeled cRNA was prepared from 100 ng of RNA using a One-color Low Input Quick Amp Labeling kit (Agilent Technologies) according to the manufacturer’s instructions, and the RNA was purified using an RNeasy Mini kit (Qiagen). Dye incorporation and the cRNA yield were evaluated using the Nanodrop 2000 spectrophotometer. Cy3-labeled cRNA was hybridized to SurePrint G3 Human GE version 2.0 containing 50,599 probes (Agilent Technologies) for 17 h at 65 °C while rotating in an Agilent hybridization oven. After hybridization, the microarrays were washed for 1 min at room temperature with GE Wash Buffer 1 (Agilent Technologies) and for 1 min at 37 °C with GE Wash Buffer 2 (Agilent Technologies). The microarrays were then dried using the Agilent stabilization and drying solution. The slides were scanned using an Agilent DNA microarray scanner immediately after washing [28]. The scanned images were quantitated using GeneSpring version 13.1.1 software (Agilent Technologies) to generate raw signal intensity data. The raw signals were log-transformed and normalized (GeneSpring software). The difference in the normalized microarray signal intensities (fold change) between the tumor and adjacent normal tissue were then calculated [29].

## Results

We used WES to analyze 18,835 genes in paired tumor tissue and blood samples to detect genetic changes in 19 different tumors. Simultaneously, we used the CCP comprising 409 target genes to conduct deep sequencing of tumor tissue samples. The mean depth of coverage of the target regions was 118-fold for WES and 1,101-fold for the CCP. We detected the following 9,439 non-synonymous single nucleotide variants (SNVs) by WES and CCP using 409 target genes in 907 patient tumors: 6,889 missense, 858 nonsense, 229 splice site, 1309 frameshift, and 154 other mutations. The genes listed in Supplementary Table 2 are classified as oncogenes or tumor suppressor genes according to Vogelstein et al. [30]. If there were multiple mutations found in a gene, then all of the mutations were counted. There are 30 genes, including *BRCA1* and *BRCA2*, that are not involved in the CCP (the genes are marked by an asterisk in Supplementary Table 2). The non-synonymous SNVs of well-annotated cancer genes, such as *PIK3CA, APC, KRAS, CTNNB1, FBXW7, GATA3 and VHL*, and *TP53*, were consistent with those of previous studies [3, 4, 31].

Somatic mutations in *TP53* were the most frequently detected (52.7%) in the set of cancer-related genes. The frequencies of missense, nonsense, frameshift, and splice site somatic mutations in *TP53* were 72.0, 14.2, 8.2, and 5.6%, respectively. The tumor frequencies were the following: colorectum (72.0%), esophagus (61.1%), stomach (59.5%), head and neck (57.5%), lung (48.9%), and pancreas (38.9%) (Table 2). There were no *TP53* mutations detected in renal cancer, melanoma, thymic tumor, or gastrointestinal stromal tumor (GIST). The data indicate that 92.5% of the somatic mutations were identified in the DNA-binding domain of TP53.

**Table 2:** Frequencies of non-synonymous somatic mutations in members of the *TP53* family and its related genes

Cancer types	*n*	*TP53* (%)	*TP63* (%)	*TP73* (%)	*TP53BP1* (%)	*TP53BP2* (%)	*TP53I3* (%)	*TP53I11* (%)	*TP53I13* (%)	*TP53AIP1* (%)	*TP53TG5* (%)	*TP53INP2* (%)	*TP53RK* (%)
Stomach	116	59.5	1.7	5.2	2.6	0.9	0	0	0	0	0	0	0
Lung	176	48.9	1.1	0	2.8	0	0.6	0.6	0	0.6	0	0.6	0
Colorectum	311	72.0	2.3	1.6	2.6	1.3	0	0	0.6	0.3	0.3	0	0.3
Breast	60	28.3	0	0	0	0	0	0	0	0	0	0	0
Liver	61	19.7	1.6	1.6	1.6	1.6	0	0	0	0	1.6	0	0
Head and neck	73	57.5	1.4	0	0	2.7	0	0	0	0	0	0	0
Pancreas	18	38.9	0	0	0	0	0	0	0	0	0	0	0
Kidney	13	0	0	0	0	0	0	0	0	0	0	0	0
Esophagus	18	61.1	0	0	0	0	0	0	0	0	0	0	0
Uterus	12	16.7	8.3	0	8.3	8.3	0	0	0	0	0	0	0
Sarcoma	16	18.8	0	0	6.3	0	0	0	0	0	0	0	0
GIST	9	0	0	0	0	0	0	0	0	0	0	0	0
Melanoma	5	0	0	0	0	0	0	0	0	0	0	0	0
Thymus	6	0	0	0	0	0	0	0	0	0	0	0	0
Ovary	4	25.0	0	0	0	0	0	0	0	0	0	0	0

The frequencies of somatic mutations in members of the *TP53* family and its related genes were low (Table 2). However, we detected increased frequencies of somatic mutations among genes encoding components of the TP53 signaling pathway (Table 3). These genes are important and well-established genes for p53-associated responses [8, 9]. The mutation data include the following: *PTEN* (11.7 and 8.7%) in breast and colorectal cancer; *ATM* (18.0 and 11.1%) in liver and esophagus cancer; *CDKN2A* (11.1 and 9.6%) in pancreas and head and neck cancer; and *ATM* (50.0%), *ATR* (41.7%), *PTEN* (83.3%), *RB1* (41.7%), and *EP300* (33.3%), which is an acetyltransferase (HAT) associated with TP53 acetylation [32], in uterine cancer. We detected the wild-type pleckstrin homology-like domain family member 3 (*PHLDA3*), which is a TP53-regulated repressor of AKT [33], and the TP53-upregulated modulator of apoptosis (*PUMA*) in all samples.

**Table 3:** Frequencies of non-synonymous somatic mutations in TP53 pathway-associated genes

Cancer types	*n*	*ATM* (%)	*ATR* (%)	*PTEN* (%)	*RB1* (%)	*CDKN1A* (%)	*CDKN2A* (%)	*MDM2* (%)	*AKT1 *(%)	*BAX* (%)	*CCND1* (%)	*CCNE1 *(%)	*PHLDA3* (%)	*PUMA* (%)	*CREBBP *(%)	*EP300 *(%)
Stomach	116	5.2	2.6	6.0	4.3	0	2.6	0	2.6	0	1.7	0	0	0	5.2	6.0
Lung	176	5.1	1.7	4.0	5.1	0.6	1.7	0.6	0	0.6	2.3	0.6	0	0	4.5	2.8
Colorectum	311	8.7	2.3	8.7	1.6	0.3	0.6	0.3	1	0.3	0.6	0.3	0	0	5.1	3.5
Breast	60	3.3	5.0	11.7	1.7	0	0	0	6.7	0	0	1.7	0	0	0	3.3
Liver	61	18.0	0	0	3.3	0	1.6	0	3.3	0	0	0	0	0	3.3	1.6
Head and neck	73	2.7	5.5	4.1	0	1.4	9.6	1.4	0	0	0	0	0	0	4.1	2.7
Pancreas	18	0	0	0	0	0	11.1	0	0	0	0	0	0	0	5.6	0
Kidney	13	0	7.7	0	0	0	0	0	0	0	7.7	0	0	0	7.7	7.7
Esophagus	18	11.1	0	0	0	0	5.6	0	0	0	0	0	0	0	0	5.6
Uterus	12	50.0	41.7	83.3	41.7	0	8.3	0	0	0	8.3	0	0	0	8.3	33.3
Sarcoma	16	0	0	0	0	0	0	0	0	0	0	0	0	0	0	0
GIST	9	0	0	0	0	0	0	0	0	0	0	0	0	0	0	0
Melanoma	5	0	0	0	0	0	0	0	0	0	0	0	0	0	0	0
Thymus	6	0	0	0	0	0	0	0	0	0	0	0	0	0	0	0
Ovary	4	0	0	0	0	0	0	0	0	0	0	0	0	0	0	25.0

Possible interactions between the *TP53*-related mutations and smoking status were examined in the stratified analyses (Supplementary Table 3). Among them, the *TP53* mutation in smoking status was found to be associated with lung cancers in a statistically significant manner (*P* = .0169). One limitation of the present study is that we had insufficient information on the drinking status of the enrolled subjects. Possible interactions with smoking status, and other environmental/lifestyle-related factors need to be evaluated in further studies.

**Fig. 1:**
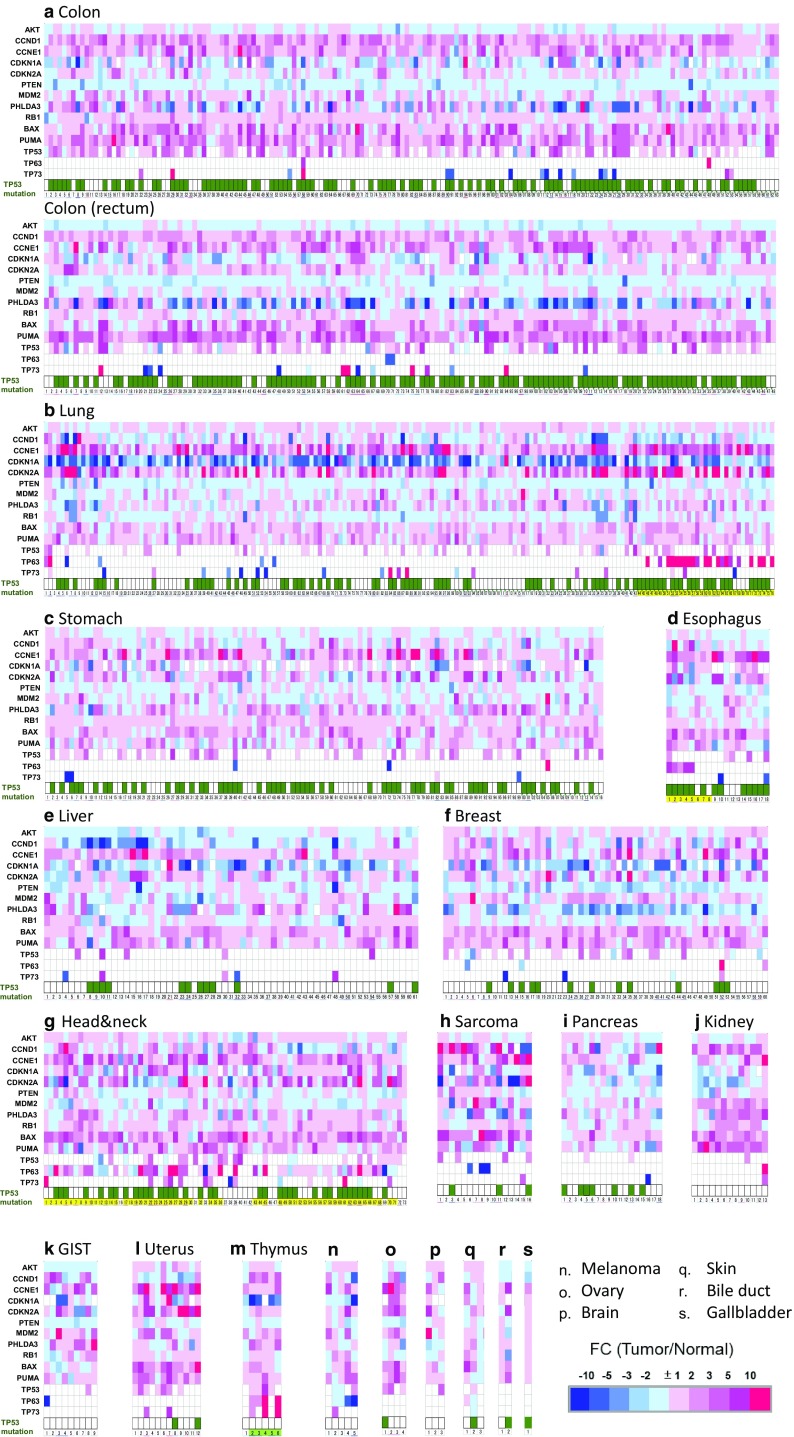
Analysis of gene expression profiles of 19 tumor types. Heat map showing 14 of the TP53 pathway-associated genes that were differentially expressed in tumor tissues relative to adjacent normal tissues. The expression levels (log_2_) were normalized for each gene and are shown by the graded color scale, with *red* and *blue* representing high and low expressions, respectively. *White squares* indicate the expression levels (absent call) for which the fold change (FC) could not be calculated, as described in Methods. *TP53* status (*bottom*) is indicated by *dark green* and *white squares* that indicate the presence and absence of mutations, respectively. *Yellow bars* with numbers beneath the graphs indicate squamous cell carcinomas of the lung, esophagus, and head and neck region. *Bright green bars with numbers* (*bottommost*) indicate thymoma cases in thymus. **a** Colon (*n* = 163), rectum (*n* = 148); **b** lung (*n* = 176); **c** stomach (*n* = 116); **d** esophagus (*n* = 18); **e** liver (*n* = 61); **f** breast (*n* = 60); **g** head & neck (*n* = 73); **h** sarcoma (*n* = 16); **i** pancreas (*n* = 18); **j** kidney (*n* = 13); **k** GIST (*n* = 9); **l** uterus (*n* = 12); **m** thymus (*n* = 6); **n** melanoma (*n* = 5); **o** ovary (*n* = 4); **p** brain (*n* = 3); **q** skin (*n* = 3); **r** bile duct (*n* = 2); **s** gallbladder (*n* = 1). The *bottommost* number shows an individual tumor

We next used microarrays to conduct gene expression profiling analysis on pairs of tumors and adjacent normal tissue (Fig. 1). The following genes were overexpressed in various tumors: *CCND1* in colorectal and renal cancers, and sarcoma; *CCNE1* in colorectal, lung, stomach, esophagus, head and neck, uterine and ovarian cancers, and sarcoma; and *CDKN2A* in lung, uterine, and ovarian cancers. *PHLDA3* expression was decreased in breast and rectal cancer. However, *PHLDA3* was increased in renal cancer and GIST. The expression level of *AKT1* was decreased in renal cancer and GIST. The expressions *BAX* and *PUMA* were increased in the majority of samples. *TP53* overexpression was detected in colorectal cancer and *TP63* overexpression was characteristically detected in squamous cell carcinoma of the lung, esophagus, and tumors in the head and neck region. Moreover, the expression levels of *TP53, TP63*, and *TP73* were increased at high frequency in thymomas.

**Fig. 2:**
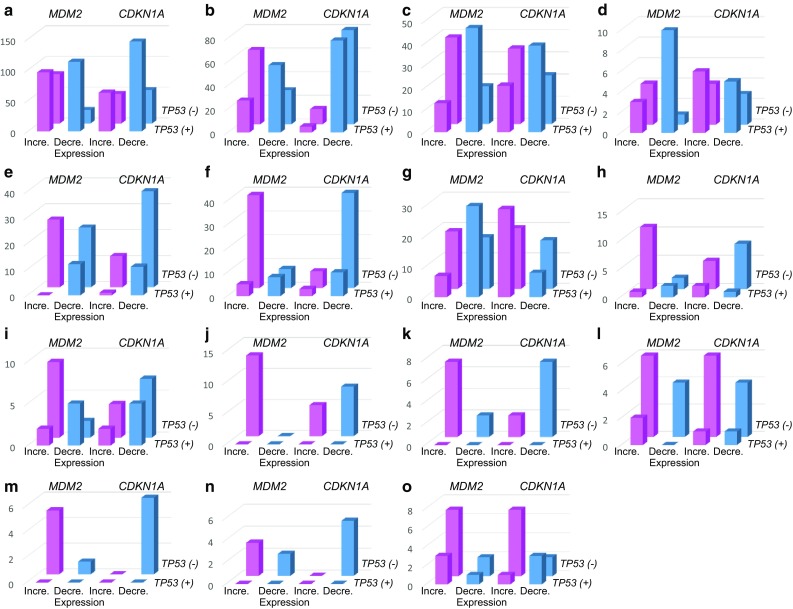
Correlations between the *TP53* status and *MDM2* or *CDKN1A* expression levels in various tumor types. The *TP53* status is indicated as mutated (+) or non-mutated (−). In each case, the *MDM2* or *CDKN1A* expression level is denoted as the number of tumor tissues with increased (*red*) or decreased (*blue*) expression relative to adjacent normal tissues, as described in Methods. **a** Colorectum (*n* = 311); **b** lung (*n* = 176); **c** stomach (*n* = 116); **d** esophagus (*n* = 18); **e** liver (*n* = 61); **f** breast (*n* = 60); **g** head & neck (*n* = 73); **h** sarcoma (*n* = 16); **i** pancreas (*n* = 18); **j** kidney (*n* = 13); **k** GIST (*n* = 9); **l** uterus (*n* = 12); **m** thymus (*n* = 6); **n** melanoma (*n* = 5); **o** others (*n* = 13). Others were as follows: ovary (*n* = 4), brain (*n* = 3), bile duct (*n* = 2), skin (*n* = 3), and gallbladder (*n* = 1). Somatic *TP53* mutations were not detected in kidney, GIST, thymus, and melanoma

We compared the expression levels of the most important TP53-responsive genes *MDM2* and *CDKN1A* (encoding p21) based on *TP53* status (Fig. 2). This analysis indicated that *MDM2* was consistently expressed at a high level in the surgical specimens of renal cancer, thymic tumor, and GIST. However, somatic mutations in *TP53* were not detected. In patients with other cancer types, the absence of a somatic mutation in *TP53* was commonly associated with increased *MDM2* expression, except colorectal cancers. In contrast, the presence of somatic mutations in *TP53* was associated with decreased *MDM2* expression. While the expression levels of *CDKN1A* were increased in surgical specimens from the esophagus and head and neck cancers with a mutated TP53 gene, the expression levels were unrelated to the TP53 status in patients with other cancer types.

## Discussion

Genes encoding downstream components of the TP53 signaling pathway were identified in studies using various inducible promoters in cancer cell lines, gene silencing, and transgenic knock-in models [34]. Additionally, recent extensive cancer genome analyses have revealed that numerous genes encoding components of the TP53 pathway are altered in human cancers. These findings suggest that the TP53 pathway plays a critical role in a range of malignancies [9]. These are currently a limited number of studies examining gene expression simultaneously in fresh tissues from multiple tumor types in a Japanese population to determine *TP53* status or mutations in genes encoding components of the pathway.

In the present study, we detected *TP53* mutations and other genetic abnormalities in the TP53 pathway in many tumors. We were intrigued that our microarray analysis revealed that *MDM2* was frequently expressed at high levels in patients with wild-type *TP53*. We assume in these patients that MDM2 formed a complex with wild-type *TP5*3 and inhibited the ability of TP53 to activate transcription of its target gene(s). The overexpression of *MDM2* promotes cell proliferation and tumorigenesis and is correlated with poor clinical outcomes [35]. The inactivation of MDM2 is essential for the activation of TP53. Thus, MDM2 may represent an independent target for drug development. For example, Tovar et al. [36] reported that the small molecule RG7112 acts as an MDM2 antagonist and showed potent antitumor activity in tumors expressing wild-type TP53 in xenograft mouse models. In addition, we detected *CDKN1A* overexpression in tumors of the colorectum, head and neck, esophagus, and stomach with mutated *TP53. CDKN1A* is a key regulator of the cell cycles, cell death, DNA repair, and cell motility [37]. Several studies have indicated that the *CDKN1A* overexpression is correlated with poor prognosis in different cancers, including esophageal carcinoma [38, 39]. Thus, identifying target molecules based on *TP53* status may facilitate the stratification of patients and development of more effective targeted therapies. *TP63* is frequently expressed in squamous cell carcinomas of the lung, head and neck region, and esophagus [40–42]. In this study, we detected high levels of *TP63* expression in patients with these carcinomas. Moreover, we demonstrate that *TP53, T63*, and *TP73* were frequently expressed in thymomas. There are a limited number of reports describing the gene expressions in thymoma patients [43]. *TP63* and *TP73* encode a *C*-terminal sterile-alpha-motif domain that is not present in TP53. This domain is important for protein–protein interactions and is associated with regulating development [44]. The transcription factors TP63 and TP73 are phosphorylated and play important roles in the activation of transcription genes controlling apoptosis [45]. *TP63* also has essential roles in embryogenesis and in the maintenance and differentiation of epithelial stem cells [46, 47]. *TP63* and *TP73* are overexpressed in human cancers, and their loss affects tumor progression and metastasis [45]. Moreover, abnormal splicing caused by *TP63*/*TP73* overexpression is frequently observed in human malignancies and is associated with poor clinical outcomes [44]. Thus *TP63*/*TP73* may be promising new targets for treating thymomas.

In this study, we used WES and global gene expression profiling to reveal the types of genetic abnormalities that occur in Japanese patients with cancer. Several types of cancer-acquired mechanisms result in the inactivation of the *TP53* or components of its signal transduction pathway. Thus, restoration of the TP53-mediated tumor suppression system could serve as a key strategy for preventing tumor development and progression. Understanding how target genes are involved in the TP53 pathway in many tumor types is essential for selecting patients who will respond to cancer therapy. We expect that our study will lead to further functional characterization of genes in the context of TP53-based individualized therapy.

## Electronic supplementary material

Below is the link to the electronic supplementary material.

Supplementary material 1 (PDF 366 KB)
