# Supplementary material for: Comprehensive characterization of genes associated with the TP53 signal transduction pathway in various tumors
Source: Mol Cell Biochem. 2017 Mar 3;431(1):75–85. doi: 10.1007/s11010-017-2977-1 (PMC5487743; doi:10.1007/s11010-017-2977-1)
Supplement: Supplementary file 1 — Supplementary material 1 (PDF 366 KB) [file 11010_2017_2977_MOESM1_ESM.pdf]

Supplementary materials

Comprehensive characterization of genes associated with the TP53 signal transduction pathway in various tumors

Journal name: *Molecular and Cellular Biochemistry*

Shumpei Ohnami[1], Keiichi Ohshima[2], Takeshi Nagashima[1,3], Kenichi Urakami[1], Yuji Shimoda[1,3], Junko Saito[4], Akane Naruoka[4], Keiichi Hatakeyama[2], Tohru Mochizuki[2], Masakuni Serizawa[4], Sumiko Ohnami[1], Masatoshi Kusuhara[4,5] and Ken Yamaguchi[6]

[1]Cancer Diagnostics Research Division, Shizuoka Cancer Center Research Institute

[2]Medical Genetics Division, Shizuoka Cancer Center Research Institute

[3]SRL Inc.

[4] Drug Discovery and Development Division, Shizuoka Cancer Center Research Institute

[5] Regional Resources Division, Shizuoka Cancer Center Research Institute

[6]Shizuoka Cancer Center

Corresponding author*:* Shumpei Ohnami, Cancer Diagnostics Research Division,

Shizuoka Cancer Center Research Institute, 1007 Shimonagakubo, Nagaizumi-cho,

Sunto-gun, Shizuoka 411-8777, Japan

Phone: +81-55-989-5222; Fax: +81-55-989-6085; E-mail: s. onami@scchr.jp

Supplementary Tables

Supplementary Table 1

Histopathological characteristics of patients.

Supplementary Table 2

The number of tumor-specific non-synonymous somatic mutations found in 19 tumor types identified using whole exome sequencing (n = 907).

Supplementary Table 3

Association between patient characteristics and TP53-related mutations in many type of tumors

Supplementary **Table 1** Histopathological characteristics of patients

Tumor types	Cases	Age
	n	mean± SD
**Stomach**	116	71.1± 9.3
Moderately differentiated tubular adenocarcinoma	41	
Poorly differentiated adenocarcinoma	43	
Well differentiated tubular adenocarcinoma	11	
Signet-ring cell carcinoma	4	
Mucinous adenocarcinoma	3	
Papillary adenocarcinoma	3	
Neruoendocrine carcinoma	2	
Adenosquamous carcinoma	3	
Mixed adenocarcioma and neuroendocrine carcinoma	3	
Small cell neuroendocrine carcinoma	1	
AFP-producing (hepatoid) adenocarcinoma	1	
Endocrine carcinoma	1	
**Lung**	176	68.6±9.9
Adenocarcinoma	122	
Squamous cell carcinoma	33	
Adenoid cystic carcinoma	2	
Adenosquamous carcinoma	1	
Combined small cell carcinoma and adenocarcinoma	1	
Diffuse malignant mesothelioma	1	
Enteric adenocarcinoma	1	
Large cell neuroendocrine carcinoma (LCNEC)	7	
Leiomyomatous hamartoma	1	
Schwannoma	2	
Mucinous AIS-like bronchiolar cell hyperplasia	1	
Pleomorphic carcinoma	2	
Small cell carcinoma	1	
Solitary fibrous tumor	1	
**Colon**	311	66.0±11.3
Cecum	21	
Well differentiated tubular adenocarcinoma	14	
Moderately differentiated tubular adenocarcinoma	6	
Mucinous adenocarcinoma	1	
Ascending colon	47	
Moderately differentiated tubular adenocarcinoma	19	
Well differentiated tubular adenocarcinoma	23	
Poorly differentiated adenocarcinoma	1	
Papillary adenocarcinoma	1	
Mucinous adenocarcinoma	3	
Anus	1	
Well differentiated tubular adenocarcinoma	1	

Tumor types	Cases	Age
	n	mean± SD
**Breast**	60	57.3±13.0
Ductal carcinoma	41	
Lobular carcinoma	7	
Metaplastic carcinoma	1	
Metaplastic carcinoma and invasive ductal carcinoma	1	
Metaplastic carcinoma and squamous cell carcinoma	2	
Mixed invasive cribriform carcinoma and ductal carcinoma	1	
Mixed mucinous and invasive ductal carcinoma	1	
Mucinous carcinoma	1	
Noninvasive ductal carcinoma	3	
Spindle cell carcinoma	1	
Micropapillary carcinoma	1	
**Liver**	61	70.3± 8.7
Hepatocellular carcinoma	53	
Intrahepatic cholangiocarcinoma	3	
Intrahepatic peripheral cholangiocarcinoma	1	
Combined hepatocellular and cholangiocarcinoma	3	
Cholangiocarcinoma	1	
**Pancreas**	18	67.3±15.5
Adenocarcinoma	12	
Adenosquamous carcinoma	3	
Endocrine tumor	1	
Neuroendocrine tumor	1	
Solid-pseudopapillary neoplasm	1	
**Kidney**	13	65.3±13.5
Clear cell renal cell carcinoma	6	
Chromophobe renal cell carcinoma	2	
Renal cell clear cell carcinoma	2	
Carcinoid tumor	1	
Urothelial carcinoma	1	
Papillary renal cell carcinoma	1	
**Esophagus**	18	66.5±8.9
Esophageal cancer	8	
Moderately differentiated squamous cell carcinoma	1	
Squamous cell carcinoma	7	
Esophagogastric junction cancer	10	
Well differentiated tubular adenocarcinoma	1	
Moderately differentiated adenocarcinoma	4	
Pooly differentiated adenocarcnioma	3	

Descending colon	13	
Moderately differentiated tubular adenocarcinoma	8	
Well differentiated tubular adenocarcinoma	5	
Sigmoid colon	55	
Moderately differentiated tubular adenocarcinoma	24	
Well differentiated tubular adenocarcinoma	25	
Mucinous adenocarcinoma	4	
Poorly differentiated adenocarcinoma	1	
Papillary adenocarcinoma	1	
Transverse colon	22	
Moderately differentiated tubular adenocarcinoma	12	
Mucinous adenocarcinoma	3	
Well differentiated tubular adenocarcinoma	6	
Poorly differentiated adenocarcinoma	1	
Duodenum	2	
Adenocarcinoma	2	
Jejunum	1	
Adenocarcinoma	1	
Appenndix	1	
Well differentiated tubular adenocarcinoma	1	
Recutum	148	
Moderately differentiated tubular adenocarcinoma	70	
Well differentiated tubular adenocarcinoma	75	
Poorly differentiated adenocarcinoma	3	
**Head and Neck**	73	62.5±14.8
Arytenoid fold	1	
Squamous cell carcinoma	1	
External ear canal	1	
Squamous cell carcinoma	1	
Gingiva	6	
Squamous cell carcinoma	6	
Gum	2	
Squamous cell carcinoma	2	
Larynx	5	
Squamous cell carcinoma	5	
Oropharynx	1	
Squamous cell carcinoma	1	
Thyroid gland	4	
Adenomatous goiter	1	
Papillary carcinoma	3	
Buccal mucosa	1	
Papillary adenocarcinoma	1	
AFP-producing hepatoid adenocarcinoma	1	
**Uterus**	12	55.3±12.8
Uterine body	8	
Endometrioid adenocarcinoma	7	
Clear cell adenocarcinoma	1	
Uterine cervix	4	
Adenocarcinoma	1	
Adenosquamous carcinoma	2	
Squamous cell carcinoma	1	
**Sarcoma**	16	45.4±20.4
Spindle cell sarcoma	2	
Osteosarcoma	2	
Synovial sarcoma	1	
Leiomyosarcoma	2	
Dedifferentiated liposarcoma	1	
Dermatofibrosarcoma protuberans	2	
Myxofibrosarcoma	2	
Rhabdomyosarcoma	1	
Round cell liposarcoma	1	
Chnodrosarcoma	1	
Carcinosarcoma	1	
**GIST (gastrointestinal stromal tumor)**	9	70.4±11.0
**Melanoma**	5	61.6±16.9
**Thymus**	6	63.0±9.0
Thymic carcinoma	1	
Thymoma	5	
**Ovary**	4	57.5±14.6
Clear cell adenocarcinoma	3	
Sex-cord stromal tumor	1	
**Skin**	3	52.0±14.8
Multiple neurofibroma	1	
Squamous cell carcinoma	1	
Eocine poroma	1	

Squamous cell carcinoma	1	
Cheek	3	
Squamous cell carcinoma	3	
Hard palate	1	
Acinic cell carcinoma	1	
Hypopharynx	6	
Squamous cell carcinoma	6	
Lingual root	1	
Squamous cell carcinoma	1	
Lip	1	
Squamous cell carcinoma	1	
Mandible	2	
Squamous cell carcinoma	2	
Maxillary sinus	1	
Adenoid cystic carcinoma	1	
Mouth	4	
Intermediate maignant tumor	1	
Squamous cell carcinoma	3	
Palate	1	
Pleomorphic adenoma with pseudoepitheliomatous hyperplasia	1	
Parotid gland	6	
Acinic cell carcinoma	1	
Carcinoma ex basal cell adenoma	1	
Carcinoma ex pleomorphic adenoma (Salivary duct carcinoma)	2	
Residual salivary duct carcinoma	1	
Squamous cell carcinoma	1	
Retromolar region	1	
Squamous cell carcinoma	1	
Submandibular region	1	
Squamous cell carcinoma	1	
Tongue	21	
Squamous cell carcinoma	21	
Tonsil	3	
Spindle cell carcinoma	1	
Squamous cell carcinoma	2	
**Brain**	3	58.3±8.1
Glioblastoma	1	
Low grade glioma	1	
Oligodendroglioma	1	
**Bile duct**	2	72.0±8.5
Adenocarcinoma	2	
**Gallbladder**	1	71
Adenocarcinoma	1	
Total	907	65.5±13.0

Supplementary **Table 2** The number of tumor-specific non-synonymous somatic mutations found in 19 tumor types identified using whole exome sequencing (n=907)

Gene	Esophagus	Colorectum	Stomach	Lung	Head&Neck	Pancreas	Sarcoma	Liver	Breast	Kidney	Melanoma	Thymus	GIST	Uterus	Others**	Ovary	Bile duct	Skin	Gallbladder
No. of cases	18	311	116	176	73	18	16	61	60	13	5	6	9	12	3	4	2	3	1
ABL1	2	10	6	2	2		1		1										
*ACVR1B**		3	3	1										1					
AKT1		3	3					2	4										
ALK	1	15	6	8	2									1					
AMER1		19	2	3	1		2	1						3					
APC		348	8	4	1									7			1		
AR	1	13	4	1					1			1		2					
ARID1A	4	18	17	10	3			10	4	1				12		1			
*ARID1B**		2	2					2	2					3					
ARID2	1	20	6	6	2			4		2				2					
ASXL1	1	10	1	5				4	1					3		1			
ATM	2	27	7	9	2			11	2					6					
ATRX		11	3	8	1				2					3					
*AXIN1**	1	3	3		1			4						1					
*B2M**		8	3		1									1					
BAP1	1	3	3	3										1					
BCL2		3	2		1														
*BCOR**		6	2	3	1		1												
BRAF		23	6	4	1						1			1					
*BRCA1**		6		3				1	1					2					
*BRCA2**		7	1	2	2			1	1					7					
CARD11		9	4	4				1		1			2	4	1				
*CASP8**		5	5		5			1						1				1	
CBL		6	3	5						1				1					
CCND1		2	2	4					1	1				1					
CDC73		2	2		1									3					
CDH1		4	13	2				1	3					1					
CDKN2A	1	2	3	3	7	2		1						1			1		1
CDKN2C				2												1			
CEBPA								1		1									
CIC		8	9	4			1		2					4	1	1	1		
CREBBP		16	6	8	3	1		2		1				1					
*CRLF2**		1																	
CSF1R		6	2	3					1					3					
CTNNB1		12	8	8		1		18						2					
CYLD		2		2	1	1		1											
DAXX		3	3	3										4					
*DNMT1**		3	4		2			1	1							1			
DNMT3A	1	11	5	5	1			4	1				1	2					
EGFR		5	6	73	1									2					
EP300	1	9	7	5	2		1	1	2	1				5		1			
ERBB2		11	14	4					2				1	2			1		
EZH2		3	1	1					1										
FBXW7	2	58	4	2	3			1	1					2				1	
FGFR2		12	2	1				1	1										
FGFR3	2	7	4	3	4		1		1							1			
FLT3		9	2	3				1	1					2					
FOXL2		1	1																
*FUBP1**		1		1										1					

Supplementary Table 2 (Continued)

Gene	Esophagus	Colorectum	Stomach	Lung	Head&Neck	Pancreas	Sarcoma	Liver	Breast	Kidney	Melanoma	Thymus	GIST	Uterus	Brain	Ovary	Bile duct	Skin	Gallbladder
No. of cases	18	311	116	176	73	18	16	61	60	13	5	6	9	12	3	4	2	3	1
GATA1		3	2		2				1										
GATA2		3	2	2															
GATA3		8		4				1	9										
GNA11				1				1								1			
GNAQ		3	1	1										1					
GNAS		22	6	8	2			2	2					2					
*H3F3A**		1	1						2										
*HIST1H3B**				1	1														
HNF1A		4	2	4				2											
HRAS		1	3		3					1		1							
IDH1		1					1							1	1				
IDH2		1	1		2														
IKZF1		12	4	2				1	1										
JAK1		4	3	1					1					3					
JAK2	1	3	3	2				1						1					
JAK3	1	4	2	6	2			1	1					1					
KDM5C		5	4	2					1	1				2					
KDM6A		7	8	3	2			2						4					
KIT		7	5	2				1					15	3					
*KLF4**																			
KMT2C		26	18	10	6	2		9	6	1				4					
KMT2D	2	50	23	30	12	1		9	7	2			2	4			1		
KRAS		132	12	27		8	1	1						2					
*LMO1**																			
MAP2K1		3	3																
MAP2K4		4		3				2	1					3					
*MAP3K1**		4	3	2				2	3					2					
MDM2		1		1	1														
MDM4		2		2				1											
*MED12**		5	3	2	2		1							5					
MEN1		7	5	3	4		2	3		1				1					
MET		13	5	2				1	1	1				3					
MLH1		5	2		1				1					2					
MPL		4	2	3										2					
MSH2		2	2	3				1						1			1		
MSH6		8	3	5	1									4					
MYC		1			2									1					
MYCL		2	1	1										1					
MYCN		5	1	1				1											
MYD88		5																	
*NCOA3**		3	3	1				1											
*NCOR1**	1	7	5	2	1			1	1					4					
NF1	1	16	8	10	2			2	2					7					
NF2		4	1		2														
NFE2L2	2	2	2	3	1			1						1					
NKX2-1		3	3	1				2											
NOTCH1	1	5	12	10	16			1	1	1				4					
NOTCH2	1	11	6	6	4			1						3					
NPM1		1	1							1									

Supplementary Table 2 (Continued)

Gene	Esophagus	Colorectum	Stomach	Lung	Head&Neck	Pancreas	Sarcoma	Liver	Breast	Kidney	Melanoma	Thymus	GIST	Uterus	Brain	Ovary	Bile duct	Skin	Gallbladder
No. of cases	18	311	116	176	73	18	16	61	60	13	5	6	9	12	3	4	2	3	1
NRAS		8	1																
PAX5		3	2	5	1			1											
PBRM1		7	7	2	1		1	5		4				3					
PDGFRA		12	3					2						5					
*PHF6**																			
PIK3CA	4	49	12	8	9		3	2	21					11					
PIK3R1		14	4	3	1		2	1	2					4					
PPP2R1A		8		1	1			1											
PRDM1		1	1	1															
PTCH1	2	13	5	8	3			2						5					
PTEN		27	7	7	3				7					10					
PTPN11		5		3	1														
RB1		5	4	9			1	2	2					5					
RET	1	14	5	8	3				2					1					
*RNF43**	1	10	4	1										1					
RUNX1		6	1						1					1					
*SETBP1**		7	2	7	1			2	2					2					
SETD2		4		5				3		2		1		3					
SF3B1		3	7	4	2				2	1									
*SKP2**		1		1					1	1									
SMAD2		12	1	2															
SMAD4	1	22	9	2	1														
SMARCA4		21	17	11	2		2	2						2		1			
SMARCB1		5	1	1	2									1					
SMO		3	2	1	1		1												
*SOCS1**																			
*SOX9**		9	3	1												1			
*SPOP**			1	1										2					
*SRSF2**			1		1														
*STAG2**			1		1				2					4					
STK11		3	2	7				1						1					
TET2		10	4	3	1			2	1			1		4					
TNFAIP3		2		1					1										
TP53	11	224	69	86	42	7	3	12	14					2		1	1	1	2
*TRAF7**		3	2		1		1							1					
TSC1		7	3	4				2						1					
TSHR		7	2	1	1									1					
*U2AF1**			1	3	1									2					
VHL		1								9									
WT1		5	2	1	1				1	9				1					

Nonsynonymous somatic mutations included missense, nonsense, frame-shift insertion or deletion (indel) and splice-site mutations.
In each case, if multiple mutations were found in a gene, all were counted.
*An asterisk denotes gene that is not involovd in CCP genes

Supplementary Table 3 Association between patient characteristics and *TP53* -related mutations in many type of tumors

**Stomach**	Cases (n)	*TP53*	*TP63*	*TP73*	*TP53BP1*	*TP53BP2*	*TP53I3*	*TP53I11*	*TP53I13*	*TP53AIP1*	*TP53TG5*	*TP53INP2*	*TP53RK*	*ATM*	*ATR*	*PTEN*	*RB1*	*CDKN1A*	*CDKN2A*	*MDM2*	*AKT1*	*BAX*	*CCND1*	*CCNE1*	*PHLDA3*	*PUMA (BBC3)*	*CREBBP*	*EP300*
Total (cases)	113	52	3	6	4	1	0	0	0	2	0	1	0	5	3	3	2	0	2	0	2	0	0	0	0	0	5	4
Female	33	13	1	3	2	1	0	0	0	0	0	0	0	1	3	2	1	0	0	0	0	0	0	0	0	0	2	0
Male	80	39	2	3	2	0	0	0	0	2	0	1	0	4	0	1	1	0	2	0	2	0	0	0	0	0	3	4
P -value*		0.411	1	0.356	0.579	n.a.	n.a.	n.a.	n.a.	1	n.a.	n.a.	n.a.	1	**0.0233**	0.204	0.501	n.a.	1	n.a.	1	n.a.	n.a.	n.a.	n.a.	n.a.	0.628	0.320
>70	60	25	2	4	2	1	0	0	0	2	0	1	0	3	2	0	1	0	2	0	1	0	0	0	0	0	4	4
<=70	53	27	1	2	2	0	0	0	0	0	0	0	0	2	1	3	1	0	0	0	1	0	0	0	0	0	1	0
P -value		0.350	1	0.683	1	n.a.	n.a.	n.a.	n.a.	0.498	n.a.	n.a.	n.a.	1	1	0.1001	1	n.a.	0.498	n.a.	1	n.a.	n.a.	n.a.	n.a.	n.a.	0.369	0.121
Non-smoker	32	12	2	2	2	0	0	0	0	1	0	0	0	2	2	2	1	0	0	0	1	0	0	0	0	0	2	0
Smoker	81	40	1	4	2	1	0	0	0	1	0	1	0	3	1	1	1	0	2	0	1	0	0	0	0	0	3	4
P -value		0.298	0.193	1	0.318	n.a.	n.a.	n.a.	n.a.	0.49	n.a.	n.a.	n.a.	0.621	0.193	0.193	0.488	n.a.	1	n.a.	0.488	n.a.	n.a.	n.a.	n.a.	n.a.	0.621	0.576
Non-diabetes	94	40	1	6	4	1	0	0	0	1	0	0	0	5	3	2	2	0	2	0	2	0	0	0	0	0	4	0
Diabetes	19	12	2	0	0	0	0	0	0	1	0	1	0	0	0	1	0	0	0	0	0	0	0	0	0	0	1	4
P -value		0.131	0.0728	0.587	1	n.a.	n.a.	n.a.	n.a.	0.309	n.a.	n.a.	n.a.	0.587	1	0.428	1	n.a.	1	n.a.	1	n.a.	n.a.	n.a.	n.a.	n.a.	1	**0.0006**
Information missing**	3																											
**Lung**																												
Total (cases)	174	76	1	0	4	0	1	1	0	1	0	0	0	8	2	7	7	1	2	1	0	1	1	1	0	0	7	1
Female	62	21	0	0	1	0	0	0	0	1	0	0	0	2	0	2	0	0	0	1	0	0	0	0	0	0	1	1
Male	112	55	1	0	3	0	1	1	0	0	0	0	0	6	2	5	7	1	2	0	0	1	1	1	0	0	6	0
P -value		0.0572	n.a.	n.a.	1	n.a.	n.a.	n.a.	n.a.	n.a.	n.a.	n.a.	n.a.	0.713	0.539	1	0.0514	n.a.	0.539	n.a.	n.a.	n.a.	n.a.	n.a.	n.a.	n.a.	0.424	n.a.
>70	84	32	1	0	2	0	0	0	0	1	0	0	0	3	0	5	4	0	2	0	0	1	0	1	0	0	5	1
<=70	90	44	0	0	2	0	1	1	0	0	0	0	0	5	2	2	3	1	0	1	0	0	1	0	0	0	2	0
P -value		0.170	n.a.	n.a.	1	n.a.	n.a.	n.a.	n.a.	n.a.	n.a.	n.a.	n.a.	0.721	0.498	0.265	0.713	n.a.	0.232	n.a.	n.a.	n.a.	n.a.	n.a.	n.a.	n.a.	0.265	n.a.
Non-smoker	49	14	0	0	1	0	0	0	0	1	0	0	0	1	0	0	0	0	0	0	0	0	0	0	0	0	0	1
Smoker	125	62	1	0	3	0	1	1	0	0	0	0	0	7	2	7	7	1	2	1	0	1	1	1	0	0	7	0
P -value		**0.0169**	n.a.	n.a.	1	n.a.	n.a.	n.a.	n.a.	n.a.	n.a.	n.a.	n.a.	0.444	1	0.193	0.193	n.a.	1	n.a.	n.a.	n.a.	n.a.	n.a.	n.a.	n.a.	0.19	n.a.
Non-diabetes	145	61	1	0	3	0	1	1	0	0	0	0	0	4	2	7	7	1	2	1	0	0	1	1	0	0	5	1
Diabetes	29	15	0	0	1	0	0	0	0	1	0	0	0	4	0	0	0	0	0	0	0	1	0	0	0	0	2	0
P -value		0.413	n.a.	n.a.	0.521	n.a.	n.a.	n.a.	n.a.	n.a.	n.a.	n.a.	n.a.	**0.0273**	1	0.602	0.602	n.a.	1	n.a.	n.a.	n.a.	n.a.	n.a.	n.a.	n.a.	0.330	n.a.
Information missing	2																											
**Colorectum**																												
Total (cases)	306	192	7	5	9	3	1	0	1	1	1	0	1	16	6	10	4	1	1	1	3	1	0	1	0	0	15	7
Female	124	74	3	3	3	1	0	0	1	0	0	0	1	6	2	3	3	1	1	0	1	1	0	1	0	0	9	3
Male	182	118	4	2	6	2	1	0	0	1	1	0	0	10	4	7	1	0	0	1	2	0	0	0	0	0	6	4
P -value		0.400	1	0.398	0.743	1	n.a.	n.a.	n.a.	n.a.	n.a.	n.a.	n.a.	1	1	0.745	0.307	n.a.	n.a.	n.a.	1	n.a.	n.a.	n.a.	n.a.	n.a.	0.176	1
>70	115	75	3	3	5	2	1	0	0	0	0	0	1	5	2	3	3	0	1	1	2	0	0	0	0	0	9	2
<=70	191	117	4	2	4	1	0	0	1	1	1	0	0	11	4	7	1	1	0	0	1	1	0	1	0	0	6	5
P -value		0.542	1	0.368	0.304	0.559	n.a.	n.a.	n.a.	n.a.	n.a.	n.a.	n.a.	0.792	1	0.748	0.151	n.a.	n.a.	n.a.	0.5587	n.a.	n.a.	n.a.	n.a.	n.a.	0.0982	0.715
Non-smoker	123	73	5	1	4	1	0	0	1	1	0	0	1	5	1	3	2	1	1	0	0	1	0	1	0	0	7	5
Smoker	183	119	2	4	5	2	1	0	0	0	1	0	0	11	5	7	2	0	0	1	3	0	0	0	0	0	8	2
P -value		0.336	0.122	0.652	1	1	n.a.	n.a.	n.a.	n.a.	n.a.	n.a.	n.a.	0.603	0.407	0.745	1	n.a.	n.a.	n.a.	0.2765	n.a.	n.a.	n.a.	n.a.	n.a.	0.601	0.122
Non-diabetes	260	159	7	5	7	3	1	0	1	1	1	0	1	13	6	10	4	1	1	1	3	1	0	1	0	0	13	7
Diabetes	46	33	0	0	2	0	0	0	0	0	0	0	0	3	0	0	0	0	0	0	0	0	0	0	0	0	2	0
P -value		0.19	0.600	1	0.629	1	n.a.	n.a.	n.a.	n.a.	n.a.	n.a.	n.a.	0.717	0.596	0.369	1	n.a.	n.a.	n.a.	1	n.a.	n.a.	n.a.	n.a.	n.a.	1	0.600
Information missing	5																											
**Breast**																												
Total (cases)	60	10	0	0	0	0	0	0	0	0	0	0	0	1	1	5	0	0	0	0	3	0	0	1	0	0	0	1
Female	60	10	0	0	0	0	0	0	0	0	0	0	0	1	1	5	0	0	0	0	3	0	0	1	0	0	0	1
Male	0	0	0	0	0	0	0	0	0	0	0	0	0	0	0	0	0	0	0	0	0	0	0	0	0	0	0	0
P -value		1	n.a.	n.a.	n.a.	n.a.	n.a.	n.a.	n.a.	n.a.	n.a.	n.a.	n.a.	n.a.	n.a.	1	n.a.	n.a.	n.a.	n.a.	1	n.a.	n.a.	n.a.	n.a.	n.a.	n.a.	n.a.
>70	11	1	0	0	0	0	0	0	0	0	0	0	0	0	1	1	0	0	0	0	0	0	0	0	0	0	0	0
<=70	49	9	0	0	0	0	0	0	0	0	0	0	0	1	0	4	0	0	0	0	3	0	0	1	0	0	0	1
P -value		0.671	n.a.	n.a.	n.a.	n.a.	n.a.	n.a.	n.a.	n.a.	n.a.	n.a.	n.a.	n.a.	n.a.	1	n.a.	n.a.	n.a.	n.a.	1	n.a.	n.a.	n.a.	n.a.	n.a.	n.a.	n.a.
Non-smoker	47	6	0	0	0	0	0	0	0	0	0	0	0	1	0	2	0	0	0	0	2	0	0	1	0	0	0	1
Smoker	13	4	0	0	0	0	0	0	0	0	0	0	0	0	1	3	0	0	0	0	1	0	0	0	0	0	0	0
P -value		0.201	n.a.	n.a.	n.a.	n.a.	n.a.	n.a.	n.a.	n.a.	n.a.	n.a.	n.a.	n.a.	n.a.	0.063	n.a.	n.a.	n.a.	n.a.	0.526	n.a.	n.a.	n.a.	n.a.	n.a.	n.a.	n.a.
Non-diabetes	58	9	0	0	0	0	0	0	0	0	0	0	0	1	1	5	0	0	0	0	2	0	0	1	0	0	0	0
Diabetes	2	1	0	0	0	0	0	0	0	0	0	0	0	0	0	0	0	0	0	0	1	0	0	0	0	0	0	1
P -value		0.308	n.a.	n.a.	n.a.	n.a.	n.a.	n.a.	n.a.	n.a.	n.a.	n.a.	n.a.	n.a.	n.a.	1	n.a.	n.a.	n.a.	n.a.	0.098	n.a.	n.a.	n.a.	n.a.	n.a.	n.a.	n.a.
**Liver**																												
Total (cases)	60	11	1	1	1	1	0	0	1	0	1	0	0	8	0	0	1	0	1	0	1	0	0	0	0	0	2	1
Female	12	2	0	0	0	0	0	0	0	0	1	0	0	2	0	0	0	0	0	0	0	0	0	0	0	0	0	0
Male	48	9	1	1	1	1	0	0	1	0	0	0	0	6	0	0	1	0	1	0	1	0	0	0	0	0	2	1
P -value		1	n.a.	n.a.	n.a.	n.a.	n.a.	n.a.	n.a.	n.a.	n.a.	n.a.	n.a.	0.655	n.a.	n.a.	n.a.	n.a.	n.a.	n.a.	n.a.	n.a.	n.a.	n.a.	n.a.	n.a.	1	n.a.
>70	34	3	0	1	1	1	0	0	1	0	1	0	0	5	0	0	1	0	1	0	0	0	0	0	0	0	1	1
<=70	26	8	1	0	0	0	0	0	0	0	0	0	0	3	0	0	0	0	0	0	1	0	0	0	0	0	1	0
P -value		**0.0437**	n.a.	n.a.	n.a.	n.a.	n.a.	n.a.	n.a.	n.a.	n.a.	n.a.	n.a.	1	n.a.	n.a.	n.a.	n.a.	n.a.	n.a.	n.a.	n.a.	n.a.	n.a.	n.a.	n.a.	1	n.a.
Non-smoker	13	1	0	0	0	0	0	0	0	0	1	0	0	4	0	0	0	0	0	0	0	0	0	0	0	0	0	0
Smoker	47	10	1	1	1	1	0	0	1	0	0	0	0	4	0	0	1	0	1	0	1	0	0	0	0	0	2	1
P -value		0.427	n.a.	n.a.	n.a.	n.a.	n.a.	n.a.	n.a.	n.a.	n.a.	n.a.	n.a.	0.059	n.a.	n.a.	n.a.	n.a.	n.a.	n.a.	n.a.	n.a.	n.a.	n.a.	n.a.	n.a.	1	n.a.
Non-diabetes	48	9	1	1	1	1	0	0	1	0	1	0	0	7	0	0	1	0	1	0	1	0	0	0	0	0	2	1
Diabetes	12	2	0	0	0	0	0	0	0	0	0	0	0	1	0	0	0	0	0	0	0	0	0	0	0	0	0	0
P -value		1	n.a.	n.a.	n.a.	n.a.	n.a.	n.a.	n.a.	n.a.	n.a.	n.a.	n.a.	1	n.a.	n.a.	n.a.	n.a.	n.a.	n.a.	n.a.	n.a.	n.a.	n.a.	n.a.	n.a.	1	n.a.
Information missing	1																											
**Head&Neck**																												
Total (cases)	73	32	1	0	0	2	0	0	0	0	0	0	0	2	4	2	0	1	6	1	0	0	0	0	0	0	2	2
Female	25	7	0	0	0	0	0	0	0	0	0	0	0	0	1	0	0	0	2	1	0	0	0	0	0	0	2	2
Male	48	25	1	0	0	2	0	0	0	0	0	0	0	2	3	2	0	1	4	0	0	0	0	0	0	0	0	0
P -value		0.0809	n.a.	n.a.	n.a.	0.543	n.a.	n.a.	n.a.	n.a.	n.a.	n.a.	n.a.	0.543	1	0.543	n.a.	n.a.	1	n.a.	n.a.	n.a.	n.a.	n.a.	n.a.	n.a.	0.114	0.114
>70	29	13	1	0	0	2	0	0	0	0	0	0	0	2	2	1	0	1	5	1	0	0	0	0	0	0	1	1
<=70	44	19	0	0	0	0	0	0	0	0	0	0	0	0	2	1	0	0	1	0	0	0	0	0	0	0	1	1
P -value		1	n.a.	n.a.	n.a.	0.155	n.a.	n.a.	n.a.	n.a.	n.a.	n.a.	n.a.	0.155	1	1	n.a.	n.a.	**0.0335**	n.a.	n.a.	n.a.	n.a.	n.a.	n.a.	n.a.	1	1
Non-smoker	26	8	1	0	0	1	0	0	0	0	0	0	0	1	1	1	0	0	3	1	0	0	0	0	0	0	2	2
Smoker	47	24	0	0	0	1	0	0	0	0	0	0	0	1	3	1	0	1	3	0	0	0	0	0	0	0	0	0
P -value		0.139	n.a.	n.a.	n.a.	1	n.a.	n.a.	n.a.	n.a.	n.a.	n.a.	n.a.	1	1	1	n.a.	n.a.	0.660	n.a.	n.a.	n.a.	n.a.	n.a.	n.a.	n.a.	0.124	0.124
Non-diabetes	64	26	1	0	0	1	0	0	0	0	0	0	0	2	4	2	0	1	6	1	0	0	0	0	0	0	2	2
Diabetes	9	6	0	0	0	1	0	0	0	0	0	0	0	0	0	0	0	0	0	0	0	0	0	0	0	0	0	0
P -value		0.1678	n.a.	n.a.	n.a.	0.233	n.a.	n.a.	n.a.	n.a.	n.a.	n.a.	n.a.	1	1	1	n.a.	n.a.	1	n.a.	n.a.	n.a.	n.a.	n.a.	n.a.	n.a.	1	1
**Pancreas**																												
Total (cases)	17	7	0	0	0	0	0	0	0	0	0	0	0	0	0	0	0	0	2	0	0	0	0	0	0	0	1	0
Female	6	3	0	0	0	0	0	0	0	0	0	0	0	0	0	0	0	0	0	0	0	0	0	0	0	0	1	0
Male	11	4	0	0	0	0	0	0	0	0	0	0	0	0	0	0	0	0	2	0	0	0	0	0	0	0	0	0
P -value		0.644	n.a.	n.a.	n.a.	n.a.	n.a.	n.a.	n.a.	n.a.	n.a.	n.a.	n.a.	n.a.	n.a.	n.a.	n.a.	n.a.	0.515	n.a.	n.a.	n.a.	n.a.	n.a.	n.a.	n.a.	n.a.	n.a.
>70	8	3	0	0	0	0	0	0	0	0	0	0	0	0	0	0	0	0	1	0	0	0	0	0	0	0	1	0
<=70	9	4	0	0	0	0	0	0	0	0	0	0	0	0	0	0	0	0	1	0	0	0	0	0	0	0	0	0
P -value		1	n.a.	n.a.	n.a.	n.a.	n.a.	n.a.	n.a.	n.a.	n.a.	n.a.	n.a.	n.a.	n.a.	n.a.	n.a.	n.a.	1	n.a.	n.a.	n.a.	n.a.	n.a.	n.a.	n.a.	n.a.	n.a.
Non-smoker	5	3	0	0	0	0	0	0	0	0	0	0	0	0	0	0	0	0	0	0	0	0	0	0	0	0	1	0
Smoker	12	4	0	0	0	0	0	0	0	0	0	0	0	0	0	0	0	0	2	0	0	0	0	0	0	0	0	0
P -value		0.593	n.a.	n.a.	n.a.	n.a.	n.a.	n.a.	n.a.	n.a.	n.a.	n.a.	n.a.	n.a.	n.a.	n.a.	n.a.	n.a.	1	n.a.	n.a.	n.a.	n.a.	n.a.	n.a.	n.a.	n.a.	n.a.
Non-diabetes	13	4	0	0	0	0	0	0	0	0	0	0	0	0	0	0	0	0	0	0	0	0	0	0	0	0	1	0
Diabetes	4	3	0	0	0	0	0	0	0	0	0	0	0	0	0	0	0	0	2	0	0	0	0	0	0	0	0	0
P -value		0.250	n.a.	n.a.	n.a.	n.a.	n.a.	n.a.	n.a.	n.a.	n.a.	n.a.	n.a.	n.a.	n.a.	n.a.	n.a.	n.a.	**0.044**	n.a.	n.a.	n.a.	n.a.	n.a.	n.a.	n.a.	n.a.	n.a.
Information missing	1																											

Supplementary Table 3 (continued)

Kidney

Total (cases)	13	0	0	0	0	0	0	0	0	0	0	0	0	0	1	0	0	0	0	0	0	0	0	0	0	0	1	0
Female	6	0	0	0	0	0	0	0	0	0	0	0	0	0	0	0	0	0	0	0	0	0	0	0	0	0	0	0
Male	7	0	0	0	0	0	0	0	0	0	0	0	0	0	1	0	0	0	0	0	0	0	0	0	0	0	1	0
P-value		n.a.	n.a.	n.a.	n.a.	n.a.	n.a.	n.a.	n.a.	n.a.	n.a.	n.a.	n.a.	n.a.	n.a.	n.a.	n.a.	n.a.	n.a.	n.a.	n.a.	n.a.	n.a.	n.a.	n.a.	n.a.	n.a.	n.a.
>70	5	0	0	0	0	0	0	0	0	0	0	0	0	0	1	0	0	0	0	0	0	0	0	0	0	0	0	0
<=70	8	0	0	0	0	0	0	0	0	0	0	0	0	0	0	0	0	0	0	0	0	0	0	0	0	0	1	0
P-value		n.a.	n.a.	n.a.	n.a.	n.a.	n.a.	n.a.	n.a.	n.a.	n.a.	n.a.	n.a.	n.a.	n.a.	n.a.	n.a.	n.a.	n.a.	n.a.	n.a.	n.a.	n.a.	n.a.	n.a.	n.a.	n.a.	n.a.
Non-smoker	5	0	0	0	0	0	0	0	0	0	0	0	0	0	0	0	0	0	0	0	0	0	0	0	0	0	0	0
Smoker	8	0	0	0	0	0	0	0	0	0	0	0	0	0	1	0	0	0	0	0	0	0	0	0	0	0	1	0
P-value		n.a.	n.a.	n.a.	n.a.	n.a.	n.a.	n.a.	n.a.	n.a.	n.a.	n.a.	n.a.	n.a.	n.a.	n.a.	n.a.	n.a.	n.a.	n.a.	n.a.	n.a.	n.a.	n.a.	n.a.	n.a.	n.a.	n.a.
Non-diabetes	12	0	0	0	0	0	0	0	0	0	0	0	0	0	1	0	0	0	0	0	0	0	0	0	0	0	1	0
Diabetes	1	0	0	0	0	0	0	0	0	0	0	0	0	0	0	0	0	0	0	0	0	0	0	0	0	0	0	0
P-value		n.a.	n.a.	n.a.	n.a.	n.a.	n.a.	n.a.	n.a.	n.a.	n.a.	n.a.	n.a.	n.a.	n.a.	n.a.	n.a.	n.a.	n.a.	n.a.	n.a.	n.a.	n.a.	n.a.	n.a.	n.a.	n.a.	n.a.

Esophagus

Total (cases)	17	10	0	0	0	0	0	0	0	0	0	0	0	2	0	0	0	0	0	0	0	0	0	0	0	0	0	1
Female	3	1	0	0	0	0	0	0	0	0	0	0	0	0	0	0	0	0	0	0	0	0	0	0	0	0	0	1
Male	14	9	0	0	0	0	0	0	0	0	0	0	0	2	0	0	0	0	0	0	0	0	0	0	0	0	0	0
P-value		0.537	n.a.	n.a.	n.a.	n.a.	n.a.	n.a.	n.a.	n.a.	n.a.	n.a.	n.a.	1	n.a.	n.a.	n.a.	n.a.	n.a.	n.a.	n.a.	n.a.	n.a.	n.a.	n.a.	n.a.	n.a.	n.a.
>70	5	3	0	0	0	0	0	0	0	0	0	0	0	1	0	0	0	0	0	0	0	0	0	0	0	0	0	0
<=70	12	7	0	0	0	0	0	0	0	0	0	0	0	1	0	0	0	0	0	0	0	0	0	0	0	0	0	1
P-value		1	n.a.	n.a.	n.a.	n.a.	n.a.	n.a.	n.a.	n.a.	n.a.	n.a.	n.a.	0.515	n.a.	n.a.	n.a.	n.a.	n.a.	n.a.	n.a.	n.a.	n.a.	n.a.	n.a.	n.a.	n.a.	n.a.
Non-smoker	5	4	0	0	0	0	0	0	0	0	0	0	0	1	0	0	0	0	0	0	0	0	0	0	0	0	0	1
Smoker	12	6	0	0	0	0	0	0	0	0	0	0	0	1	0	0	0	0	0	0	0	0	0	0	0	0	0	0
P-value		0.338	n.a.	n.a.	n.a.	n.a.	n.a.	n.a.	n.a.	n.a.	n.a.	n.a.	n.a.	0.515	n.a.	n.a.	n.a.	n.a.	n.a.	n.a.	n.a.	n.a.	n.a.	n.a.	n.a.	n.a.	n.a.	n.a.
Non-diabetes	15	8	0	0	0	0	0	0	0	0	0	0	0	2	0	0	0	0	0	0	0	0	0	0	0	0	0	1
Diabetes	2	2	0	0	0	0	0	0	0	0	0	0	0	0	0	0	0	0	0	0	0	0	0	0	0	0	0	0
P-value		0.485	n.a.	n.a.	n.a.	n.a.	n.a.	n.a.	n.a.	n.a.	n.a.	n.a.	n.a.	1	n.a.	n.a.	n.a.	n.a.	n.a.	n.a.	n.a.	n.a.	n.a.	n.a.	n.a.	n.a.	n.a.	n.a.
Information missing	1																											

Uterus

Total (cases)	12	2	1	0	2	1	1	0	0	0	0	0	0	2	1	7	1	0	1	0	0	0	1	0	0	0	1	3
Female	12	2	1	0	2	1	1	0	0	0	0	0	0	2	1	7	1	0	1	0	0	0	1	0	0	0	1	3
Male	0	0	0	0	0	0	0	0	0	0	0	0	0	0	0	0	0	0	0	0	0	0	0	0	0	0	0	0
P-value		1	n.a.	n.a.	1	n.a.	n.a.	n.a.	n.a.	n.a.	n.a.	n.a.	n.a.	1	n.a.	1	n.a.	n.a.	n.a.	n.a.	n.a.	n.a.	n.a.	n.a.	n.a.	n.a.	n.a.	1
>70	2	0	0	0	0	0	0	0	0	0	0	0	0	0	0	1	0	0	0	0	0	0	0	0	0	0	0	1
<=70	10	2	1	0	2	1	1	0	0	0	0	0	0	2	1	6	1	0	1	0	0	0	1	0	0	0	1	2
P-value		1	n.a.	n.a.	1	n.a.	n.a.	n.a.	n.a.	n.a.	n.a.	n.a.	n.a.	1	n.a.	1	n.a.	n.a.	n.a.	n.a.	n.a.	n.a.	n.a.	n.a.	n.a.	n.a.	n.a.	0.455
Non-smoker	11	2	1	0	2	1	1	0	0	0	0	0	0	2	1	7	1	0	1	0	0	0	1	0	0	0	1	3
Smoker	1	0	0	0	0	0	0	0	0	0	0	0	0	0	0	0	0	0	0	0	0	0	0	0	0	0	0	0
P-value		1	n.a.	n.a.	1	n.a.	n.a.	n.a.	n.a.	n.a.	n.a.	n.a.	n.a.	1	n.a.	0.417	n.a.	n.a.	n.a.	n.a.	n.a.	n.a.	n.a.	n.a.	n.a.	n.a.	n.a.	1
Non-diabetes	12	2	1	0	2	1	1	0	0	0	0	0	0	2	1	7	1	0	1	0	0	0	1	0	0	0	1	3
Diabetes	0	0	0	0	0	0	0	0	0	0	0	0	0	0	0	0	0	0	0	0	0	0	0	0	0	0	0	0
P-value		1	n.a.	n.a.	1	n.a.	n.a.	n.a.	n.a.	n.a.	n.a.	n.a.	n.a.	1	n.a.	1	n.a.	n.a.	n.a.	n.a.	n.a.	n.a.	n.a.	n.a.	n.a.	n.a.	n.a.	1

Sarcoma

Total (cases)	15	2	0	0	1	0	0	0	0	0	0	0	0	0	0	0	0	0	0	0	0	0	0	0	0	0	0	0
Female	4	2	0	0	1	0	0	0	0	0	0	0	0	0	0	0	0	0	0	0	0	0	0	0	0	0	0	0
Male	11	0	0	0	0	0	0	0	0	0	0	0	0	0	0	0	0	0	0	0	0	0	0	0	0	0	0	0
P-value		0.0571	n.a.	n.a.	n.a.	n.a.	n.a.	n.a.	n.a.	n.a.	n.a.	n.a.	n.a.	n.a.	n.a.	n.a.	n.a.	n.a.	n.a.	n.a.	n.a.	n.a.	n.a.	n.a.	n.a.	n.a.	n.a.	n.a.
>70	4	1	0	0	0	0	0	0	0	0	0	0	0	0	0	0	0	0	0	0	0	0	0	0	0	0	0	0
<=70	11	1	0	0	1	0	0	0	0	0	0	0	0	0	0	0	0	0	0	0	0	0	0	0	0	0	0	0
P-value		0.476	n.a.	n.a.	n.a.	n.a.	n.a.	n.a.	n.a.	n.a.	n.a.	n.a.	n.a.	n.a.	n.a.	n.a.	n.a.	n.a.	n.a.	n.a.	n.a.	n.a.	n.a.	n.a.	n.a.	n.a.	n.a.	n.a.
Non-smoker	7	2	0	0	1	0	0	0	0	0	0	0	0	0	0	0	0	0	0	0	0	0	0	0	0	0	0	0
Smoker	8	0	0	0	0	0	0	0	0	0	0	0	0	0	0	0	0	0	0	0	0	0	0	0	0	0	0	0
P-value		0.200	n.a.	n.a.	n.a.	n.a.	n.a.	n.a.	n.a.	n.a.	n.a.	n.a.	n.a.	n.a.	n.a.	n.a.	n.a.	n.a.	n.a.	n.a.	n.a.	n.a.	n.a.	n.a.	n.a.	n.a.	n.a.	n.a.
Non-diabetes	15	2	0	0	1	0	0	0	0	0	0	0	0	0	0	0	0	0	0	0	0	0	0	0	0	0	0	0
Diabetes	0	0	0	0	0	0	0	0	0	0	0	0	0	0	0	0	0	0	0	0	0	0	0	0	0	0	0	0
P-value		1	n.a.	n.a.	n.a.	n.a.	n.a.	n.a.	n.a.	n.a.	n.a.	n.a.	n.a.	n.a.	n.a.	n.a.	n.a.	n.a.	n.a.	n.a.	n.a.	n.a.	n.a.	n.a.	n.a.	n.a.	n.a.	n.a.
Information missing	1																											

GIST

Total (cases)	9	0	0	0	0	0	0	0	0	0	0	0	0	0	0	0	0	0	0	0	0	0	0	0	0	0	0	0
Female	3	0	0	0	0	0	0	0	0	0	0	0	0	0	0	0	0	0	0	0	0	0	0	0	0	0	0	0
Male	6	0	0	0	0	0	0	0	0	0	0	0	0	0	0	0	0	0	0	0	0	0	0	0	0	0	0	0
P-value		n.a.	n.a.	n.a.	n.a.	n.a.	n.a.	n.a.	n.a.	n.a.	n.a.	n.a.	n.a.	n.a.	n.a.	n.a.	n.a.	n.a.	n.a.	n.a.	n.a.	n.a.	n.a.	n.a.	n.a.	n.a.	n.a.	n.a.
>70	5	0	0	0	0	0	0	0	0	0	0	0	0	0	0	0	0	0	0	0	0	0	0	0	0	0	0	0
<=70	4	0	0	0	0	0	0	0	0	0	0	0	0	0	0	0	0	0	0	0	0	0	0	0	0	0	0	0
P-value		n.a.	n.a.	n.a.	n.a.	n.a.	n.a.	n.a.	n.a.	n.a.	n.a.	n.a.	n.a.	n.a.	n.a.	n.a.	n.a.	n.a.	n.a.	n.a.	n.a.	n.a.	n.a.	n.a.	n.a.	n.a.	n.a.	n.a.
Non-smoker	6	0	0	0	0	0	0	0	0	0	0	0	0	0	0	0	0	0	0	0	0	0	0	0	0	0	0	0
Smoker	3	0	0	0	0	0	0	0	0	0	0	0	0	0	0	0	0	0	0	0	0	0	0	0	0	0	0	0
P-value		n.a.	n.a.	n.a.	n.a.	n.a.	n.a.	n.a.	n.a.	n.a.	n.a.	n.a.	n.a.	n.a.	n.a.	n.a.	n.a.	n.a.	n.a.	n.a.	n.a.	n.a.	n.a.	n.a.	n.a.	n.a.	n.a.	n.a.
Non-diabetes	7	0	0	0	0	0	0	0	0	0	0	0	0	0	0	0	0	0	0	0	0	0	0	0	0	0	0	0
Diabetes	2	0	0	0	0	0	0	0	0	0	0	0	0	0	0	0	0	0	0	0	0	0	0	0	0	0	0	0
P-value		n.a.	n.a.	n.a.	n.a.	n.a.	n.a.	n.a.	n.a.	n.a.	n.a.	n.a.	n.a.	n.a.	n.a.	n.a.	n.a.	n.a.	n.a.	n.a.	n.a.	n.a.	n.a.	n.a.	n.a.	n.a.	n.a.	n.a.

Melanoma

Total (cases)	5	0	0	0	0	0	0	0	0	0	0	0	0	0	0	0	0	0	0	0	0	0	0	0	0	0	0	0
Female	2	0	0	0	0	0	0	0	0	0	0	0	0	0	0	0	0	0	0	0	0	0	0	0	0	0	0	0
Male	3	0	0	0	0	0	0	0	0	0	0	0	0	0	0	0	0	0	0	0	0	0	0	0	0	0	0	0
P-value		n.a.	n.a.	n.a.	n.a.	n.a.	n.a.	n.a.	n.a.	n.a.	n.a.	n.a.	n.a.	n.a.	n.a.	n.a.	n.a.	n.a.	n.a.	n.a.	n.a.	n.a.	n.a.	n.a.	n.a.	n.a.	n.a.	n.a.
>70	2	0	0	0	0	0	0	0	0	0	0	0	0	0	0	0	0	0	0	0	0	0	0	0	0	0	0	0
<=70	3	0	0	0	0	0	0	0	0	0	0	0	0	0	0	0	0	0	0	0	0	0	0	0	0	0	0	0
P-value		n.a.	n.a.	n.a.	n.a.	n.a.	n.a.	n.a.	n.a.	n.a.	n.a.	n.a.	n.a.	n.a.	n.a.	n.a.	n.a.	n.a.	n.a.	n.a.	n.a.	n.a.	n.a.	n.a.	n.a.	n.a.	n.a.	n.a.
Non-smoker	4	0	0	0	0	0	0	0	0	0	0	0	0	0	0	0	0	0	0	0	0	0	0	0	0	0	0	0
Smoker	1	0	0	0	0	0	0	0	0	0	0	0	0	0	0	0	0	0	0	0	0	0	0	0	0	0	0	0
P-value		n.a.	n.a.	n.a.	n.a.	n.a.	n.a.	n.a.	n.a.	n.a.	n.a.	n.a.	n.a.	n.a.	n.a.	n.a.	n.a.	n.a.	n.a.	n.a.	n.a.	n.a.	n.a.	n.a.	n.a.	n.a.	n.a.	n.a.
Non-diabetes	4	0	0	0	0	0	0	0	0	0	0	0	0	0	0	0	0	0	0	0	0	0	0	0	0	0	0	0
Diabetes	1	0	0	0	0	0	0	0	0	0	0	0	0	0	0	0	0	0	0	0	0	0	0	0	0	0	0	0
P-value		n.a.	n.a.	n.a.	n.a.	n.a.	n.a.	n.a.	n.a.	n.a.	n.a.	n.a.	n.a.	n.a.	n.a.	n.a.	n.a.	n.a.	n.a.	n.a.	n.a.	n.a.	n.a.	n.a.	n.a.	n.a.	n.a.	n.a.

Thymus

Total (cases)	6	0	0	0	0	0	0	0	0	0	0	0	0	0	0	0	0	0	0	0	0	0	0	0	0	0	0	0
Female	5	0	0	0	0	0	0	0	0	0	0	0	0	0	0	0	0	0	0	0	0	0	0	0	0	0	0	0
Male	1	0	0	0	0	0	0	0	0	0	0	0	0	0	0	0	0	0	0	0	0	0	0	0	0	0	0	0
P-value		n.a.	n.a.	n.a.	n.a.	n.a.	n.a.	n.a.	n.a.	n.a.	n.a.	n.a.	n.a.	n.a.	n.a.	n.a.	n.a.	n.a.	n.a.	n.a.	n.a.	n.a.	n.a.	n.a.	n.a.	n.a.	n.a.	n.a.
>70	2	0	0	0	0	0	0	0	0	0	0	0	0	0	0	0	0	0	0	0	0	0	0	0	0	0	0	0
<=70	4	0	0	0	0	0	0	0	0	0	0	0	0	0	0	0	0	0	0	0	0	0	0	0	0	0	0	0
P-value		n.a.	n.a.	n.a.	n.a.	n.a.	n.a.	n.a.	n.a.	n.a.	n.a.	n.a.	n.a.	n.a.	n.a.	n.a.	n.a.	n.a.	n.a.	n.a.	n.a.	n.a.	n.a.	n.a.	n.a.	n.a.	n.a.	n.a.
Non-smoker	4	0	0	0	0	0	0	0	0	0	0	0	0	0	0	0	0	0	0	0	0	0	0	0	0	0	0	0
Smoker	2	0	0	0	0	0	0	0	0	0	0	0	0	0	0	0	0	0	0	0	0	0	0	0	0	0	0	0
P-value		n.a.	n.a.	n.a.	n.a.	n.a.	n.a.	n.a.	n.a.	n.a.	n.a.	n.a.	n.a.	n.a.	n.a.	n.a.	n.a.	n.a.	n.a.	n.a.	n.a.	n.a.	n.a.	n.a.	n.a.	n.a.	n.a.	n.a.
Non-diabetes	5	0	0	0	0	0	0	0	0	0	0	0	0	0	0	0	0	0	0	0	0	0	0	0	0	0	0	0
Diabetes	1	0	0	0	0	0	0	0	0	0	0	0	0	0	0	0	0	0	0	0	0	0	0	0	0	0	0	0
P-value		n.a.	n.a.	n.a.	n.a.	n.a.	n.a.	n.a.	n.a.	n.a.	n.a.	n.a.	n.a.	n.a.	n.a.	n.a.	n.a.	n.a.	n.a.	n.a.	n.a.	n.a.	n.a.	n.a.	n.a.	n.a.	n.a.	n.a.

Supplementary Table 3 (continud)

**Ovary**																												
Total (cases)	4	1	0	0	0	0	0	0	0	0	0	0	0	0	0	0	0	0	0	0	0	0	0	0	0	0	0	1
Female	4	1	0	0	0	0	0	0	0	0	0	0	0	0	0	0	0	0	0	0	0	0	0	0	0	0	0	1
Male	0	0	0	0	0	0	0	0	0	0	0	0	0	0	0	0	0	0	0	0	0	0	0	0	0	0	0	0
P -value		n.a.	n.a.	n.a.	n.a.	n.a.	n.a.	n.a.	n.a.	n.a.	n.a.	n.a.	n.a.	n.a.	n.a.	n.a.	n.a.	n.a.	n.a.	n.a.	n.a.	n.a.	n.a.	n.a.	n.a.	n.a.	n.a.	n.a.
>70	1	0	0	0	0	0	0	0	0	0	0	0	0	0	0	0	0	0	0	0	0	0	0	0	0	0	0	0
<=70	3	1	0	0	0	0	0	0	0	0	0	0	0	0	0	0	0	0	0	0	0	0	0	0	0	0	0	1
P -value		n.a.	n.a.	n.a.	n.a.	n.a.	n.a.	n.a.	n.a.	n.a.	n.a.	n.a.	n.a.	n.a.	n.a.	n.a.	n.a.	n.a.	n.a.	n.a.	n.a.	n.a.	n.a.	n.a.	n.a.	n.a.	n.a.	n.a.
Non-smoker	4	1	0	0	0	0	0	0	0	0	0	0	0	0	0	0	0	0	0	0	0	0	0	0	0	0	0	1
Smoker	0	0	0	0	0	0	0	0	0	0	0	0	0	0	0	0	0	0	0	0	0	0	0	0	0	0	0	0
P -value		n.a.	n.a.	n.a.	n.a.	n.a.	n.a.	n.a.	n.a.	n.a.	n.a.	n.a.	n.a.	n.a.	n.a.	n.a.	n.a.	n.a.	n.a.	n.a.	n.a.	n.a.	n.a.	n.a.	n.a.	n.a.	n.a.	n.a.
Non-diabetes	4	1	0	0	0	0	0	0	0	0	0	0	0	0	0	0	0	0	0	0	0	0	0	0	0	0	0	1
Diabetes	0	0	0	0	0	0	0	0	0	0	0	0	0	0	0	0	0	0	0	0	0	0	0	0	0	0	0	0
P -value		n.a.	n.a.	n.a.	n.a.	n.a.	n.a.	n.a.	n.a.	n.a.	n.a.	n.a.	n.a.	n.a.	n.a.	n.a.	n.a.	n.a.	n.a.	n.a.	n.a.	n.a.	n.a.	n.a.	n.a.	n.a.	n.a.	n.a.
**Skin**																												
Total (cases)	2	1	0	0	0	0	0	0	0	0	0	0	0	0	0	0	0	0	0	0	0	0	0	0	0	0	0	0
Female	1	1	0	0	0	0	0	0	0	0	0	0	0	0	0	0	0	0	0	0	0	0	0	0	0	0	0	0
Male	1	0	0	0	0	0	0	0	0	0	0	0	0	0	0	0	0	0	0	0	0	0	0	0	0	0	0	0
P -value		n.a.	n.a.	n.a.	n.a.	n.a.	n.a.	n.a.	n.a.	n.a.	n.a.	n.a.	n.a.	n.a.	n.a.	n.a.	n.a.	n.a.	n.a.	n.a.	n.a.	n.a.	n.a.	n.a.	n.a.	n.a.	n.a.	n.a.
>70	0	0	0	0	0	0	0	0	0	0	0	0	0	0	0	0	0	0	0	0	0	0	0	0	0	0	0	0
<=70	2	1	0	0	0	0	0	0	0	0	0	0	0	0	0	0	0	0	0	0	0	0	0	0	0	0	0	0
P -value		n.a.	n.a.	n.a.	n.a.	n.a.	n.a.	n.a.	n.a.	n.a.	n.a.	n.a.	n.a.	n.a.	n.a.	n.a.	n.a.	n.a.	n.a.	n.a.	n.a.	n.a.	n.a.	n.a.	n.a.	n.a.	n.a.	n.a.
Non-smoker	1	1	0	0	0	0	0	0	0	0	0	0	0	0	0	0	0	0	0	0	0	0	0	0	0	0	0	0
Smoker	1	0	0	0	0	0	0	0	0	0	0	0	0	0	0	0	0	0	0	0	0	0	0	0	0	0	0	0
P -value		n.a.	n.a.	n.a.	n.a.	n.a.	n.a.	n.a.	n.a.	n.a.	n.a.	n.a.	n.a.	n.a.	n.a.	n.a.	n.a.	n.a.	n.a.	n.a.	n.a.	n.a.	n.a.	n.a.	n.a.	n.a.	n.a.	n.a.
Non-diabetes	1	1	0	0	0	0	0	0	0	0	0	0	0	0	0	0	0	0	0	0	0	0	0	0	0	0	0	0
Diabetes	0	0	0	0	0	0	0	0	0	0	0	0	0	0	0	0	0	0	0	0	0	0	0	0	0	0	0	0
P -value		n.a.	n.a.	n.a.	n.a.	n.a.	n.a.	n.a.	n.a.	n.a.	n.a.	n.a.	n.a.	n.a.	n.a.	n.a.	n.a.	n.a.	n.a.	n.a.	n.a.	n.a.	n.a.	n.a.	n.a.	n.a.	n.a.	n.a.
Information missing	1																											
**Brain**																												
Total (cases)	3	0	0	0	0	0	0	0	0	0	0	0	0	0	0	0	0	0	0	0	0	0	0	0	0	0	0	0
Female	1	0	0	0	0	0	0	0	0	0	0	0	0	0	0	0	0	0	0	0	0	0	0	0	0	0	0	0
Male	2	0	0	0	0	0	0	0	0	0	0	0	0	0	0	0	0	0	0	0	0	0	0	0	0	0	0	0
P -value		n.a.	n.a.	n.a.	n.a.	n.a.	n.a.	n.a.	n.a.	n.a.	n.a.	n.a.	n.a.	n.a.	n.a.	n.a.	n.a.	n.a.	n.a.	n.a.	n.a.	n.a.	n.a.	n.a.	n.a.	n.a.	n.a.	n.a.
>70	0	0	0	0	0	0	0	0	0	0	0	0	0	0	0	0	0	0	0	0	0	0	0	0	0	0	0	0
<=70	3	0	0	0	0	0	0	0	0	0	0	0	0	0	0	0	0	0	0	0	0	0	0	0	0	0	0	0
P -value		n.a.	n.a.	n.a.	n.a.	n.a.	n.a.	n.a.	n.a.	n.a.	n.a.	n.a.	n.a.	n.a.	n.a.	n.a.	n.a.	n.a.	n.a.	n.a.	n.a.	n.a.	n.a.	n.a.	n.a.	n.a.	n.a.	n.a.
Non-smoker	1	0	0	0	0	0	0	0	0	0	0	0	0	0	0	0	0	0	0	0	0	0	0	0	0	0	0	0
Smoker	2	0	0	0	0	0	0	0	0	0	0	0	0	0	0	0	0	0	0	0	0	0	0	0	0	0	0	0
P -value		n.a.	n.a.	n.a.	n.a.	n.a.	n.a.	n.a.	n.a.	n.a.	n.a.	n.a.	n.a.	n.a.	n.a.	n.a.	n.a.	n.a.	n.a.	n.a.	n.a.	n.a.	n.a.	n.a.	n.a.	n.a.	n.a.	n.a.
Non-diabetes	3	0	0	0	0	0	0	0	0	0	0	0	0	0	0	0	0	0	0	0	0	0	0	0	0	0	0	0
Diabetes	0	0	0	0	0	0	0	0	0	0	0	0	0	0	0	0	0	0	0	0	0	0	0	0	0	0	0	0
P -value		n.a.	n.a.	n.a.	n.a.	n.a.	n.a.	n.a.	n.a.	n.a.	n.a.	n.a.	n.a.	n.a.	n.a.	n.a.	n.a.	n.a.	n.a.	n.a.	n.a.	n.a.	n.a.	n.a.	n.a.	n.a.	n.a.	n.a.
**Bile duct**																												
Total (cases)	2	1	0	0	0	0	0	0	0	0	0	0	0	0	0	0	0	0	1	0	0	0	0	0	0	0	0	0
Female	1	1	0	0	0	0	0	0	0	0	0	0	0	0	0	0	0	0	1	0	0	0	0	0	0	0	0	0
Male	1	0	0	0	0	0	0	0	0	0	0	0	0	0	0	0	0	0	0	0	0	0	0	0	0	0	0	0
P -value		n.a.	n.a.	n.a.	n.a.	n.a.	n.a.	n.a.	n.a.	n.a.	n.a.	n.a.	n.a.	n.a.	n.a.	n.a.	n.a.	n.a.	n.a.	n.a.	n.a.	n.a.	n.a.	n.a.	n.a.	n.a.	n.a.	n.a.
>70	1	0	0	0	0	0	0	0	0	0	0	0	0	0	0	0	0	0	0	0	0	0	0	0	0	0	0	0
<=70	1	1	0	0	0	0	0	0	0	0	0	0	0	0	0	0	0	0	1	0	0	0	0	0	0	0	0	0
P -value		n.a.	n.a.	n.a.	n.a.	n.a.	n.a.	n.a.	n.a.	n.a.	n.a.	n.a.	n.a.	n.a.	n.a.	n.a.	n.a.	n.a.	n.a.	n.a.	n.a.	n.a.	n.a.	n.a.	n.a.	n.a.	n.a.	n.a.
Non-smoker	1	1	0	0	0	0	0	0	0	0	0	0	0	0	0	0	0	0	1	0	0	0	0	0	0	0	0	0
Smoker	1	0	0	0	0	0	0	0	0	0	0	0	0	0	0	0	0	0	0	0	0	0	0	0	0	0	0	0
P -value		n.a.	n.a.	n.a.	n.a.	n.a.	n.a.	n.a.	n.a.	n.a.	n.a.	n.a.	n.a.	n.a.	n.a.	n.a.	n.a.	n.a.	n.a.	n.a.	n.a.	n.a.	n.a.	n.a.	n.a.	n.a.	n.a.	n.a.
Non-diabetes	2	1	0	0	0	0	0	0	0	0	0	0	0	0	0	0	0	0	1	0	0	0	0	0	0	0	0	0
Diabetes	0	0	0	0	0	0	0	0	0	0	0	0	0	0	0	0	0	0	0	0	0	0	0	0	0	0	0	0
P -value		n.a.	n.a.	n.a.	n.a.	n.a.	n.a.	n.a.	n.a.	n.a.	n.a.	n.a.	n.a.	n.a.	n.a.	n.a.	n.a.	n.a.	n.a.	n.a.	n.a.	n.a.	n.a.	n.a.	n.a.	n.a.	n.a.	n.a.
**Gallbladder**																												
Total (cases)	1	1	0	0	0	0	0	0	0	0	0	0	0	0	0	0	0	0	1	0	0	0	0	0	0	0	0	0
Female	1	1	0	0	0	0	0	0	0	0	0	0	0	0	0	0	0	0	1	0	0	0	0	0	0	0	0	0
Male	0	0	0	0	0	0	0	0	0	0	0	0	0	0	0	0	0	0	0	0	0	0	0	0	0	0	0	0
P -value		n.a.	n.a.	n.a.	n.a.	n.a.	n.a.	n.a.	n.a.	n.a.	n.a.	n.a.	n.a.	n.a.	n.a.	n.a.	n.a.	n.a.	n.a.	n.a.	n.a.	n.a.	n.a.	n.a.	n.a.	n.a.	n.a.	n.a.
>70	1	1	0	0	0	0	0	0	0	0	0	0	0	0	0	0	0	0	1	0	0	0	0	0	0	0	0	0
<=70	0	0	0	0	0	0	0	0	0	0	0	0	0	0	0	0	0	0	0	0	0	0	0	0	0	0	0	0
P -value		n.a.	n.a.	n.a.	n.a.	n.a.	n.a.	n.a.	n.a.	n.a.	n.a.	n.a.	n.a.	n.a.	n.a.	n.a.	n.a.	n.a.	n.a.	n.a.	n.a.	n.a.	n.a.	n.a.	n.a.	n.a.	n.a.	n.a.
Non-smoker	0	0	0	0	0	0	0	0	0	0	0	0	0	0	0	0	0	0	0	0	0	0	0	0	0	0	0	0
Smoker	1	1	0	0	0	0	0	0	0	0	0	0	0	0	0	0	0	0	1	0	0	0	0	0	0	0	0	0
P -value		n.a.	n.a.	n.a.	n.a.	n.a.	n.a.	n.a.	n.a.	n.a.	n.a.	n.a.	n.a.	n.a.	n.a.	n.a.	n.a.	n.a.	n.a.	n.a.	n.a.	n.a.	n.a.	n.a.	n.a.	n.a.	n.a.	n.a.
Non-diabetes	1	1	0	0	0	0	0	0	0	0	0	0	0	0	0	0	0	0	1	0	0	0	0	0	0	0	0	0
Diabetes	0	0	0	0	0	0	0	0	0	0	0	0	0	0	0	0	0	0	0	0	0	0	0	0	0	0	0	0
P -value		n.a.	n.a.	n.a.	n.a.	n.a.	n.a.	n.a.	n.a.	n.a.	n.a.	n.a.	n.a.	n.a.	n.a.	n.a.	n.a.	n.a.	n.a.	n.a.	n.a.	n.a.	n.a.	n.a.	n.a.	n.a.	n.a.	n.a.

*P value by Fisher exact test.

**No information about smoking status or diabetes history.

n.a.; Stastistical calculation is not applicable

Data in bold indicate statistically significant differences (P <.05)
